# Determination of antioxidant activity by in situ synthesis of AgNPs using in-tube SPME coupled on-line to capillary liquid chromatography

**DOI:** 10.1007/s00604-023-05886-w

**Published:** 2023-07-18

**Authors:** María Carmen Prieto-Blanco, María Pardo-Puñal, Yolanda Moliner-Martínez, Pilar Campíns-Falcó

**Affiliations:** 1 Grupo QANAP, Departamento de Química, Facultade de Ciencias, Instituto Universitario de Medio Ambiente (IUMA), Universidade da Coruña, Campus da Zapateira, 15071 A Coruña, Spain; 2 Grupo MINTOTA, Departament de Química Analítica, Facultat de Química, Universitat de Valencia C/ Dr. Moliner 50, E46100, Burjassot, Valencia Spain

**Keywords:** Silver nanoparticles, IT-SPME-CapLC, Chlorogenic acid, Trolox, Biosynthesis, Autocatalytic growth

## Abstract

**Abstract:**

A chromatographic system based on in-tube SPME coupled to capillary LC-DAD has been used to study the synthesis of silver nanoparticles using polyphenols in different scenarios: excess of the reducing agent or of the silver salt, addition of the cationic surfactants, and thermal synthesis. The optimized synthesis conditions allowed to quantify the polyphenols used as reducing agents, such as Trolox and chlorogenic acid. Two chromatographic peaks with different absorption spectrum were monitored during the syntheses. Depending on the molar relationship, a linear relation between the area of the chromatographic peaks and the concentration of the silver or polyphenol was established. For stabilization of silver nanoparticles, different cationic surfactants were used allowing to evaluate the role of anion (chloride and bromide) and of the alkyl chain. The proposed methodology can be used to determine chlorogenic acid up to 3 mM with a detection limit of 34 μM at λ= 400 nm. Chlorogenic acid was determined in dietary products with successful results. Precision (RSD=10%) and recovery (97–100%) were also satisfactory.

**Graphical Abstract:**

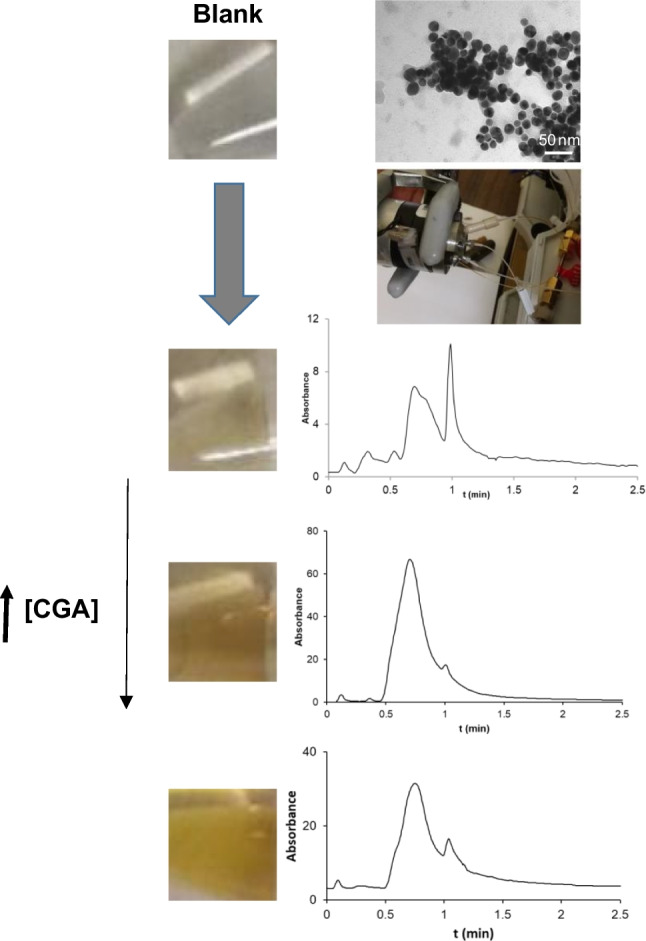

**Supplementary Information:**

The online version contains supplementary material available at 10.1007/s00604-023-05886-w.

## Introduction

Nanoparticle synthesis can be carried out using the extracts of plants or other organisms such as fungi, bacteria, and yeast [1, 2]. Some of the compound classes (polyphenols, flavonoids, and polysaccharides, among others) present in the plants may act as reducing agents of metallic salts, in particular silver salts, under specific conditions. Anti-fungal, anti-microbial, anti-protozoal, and anticancer properties have been found in silver NPs synthetized by this method [2]. Some toxic components used in conventional synthesis (solvents and strong reducing agents) are replaced by water and by more compatible environmentally reducing agents in biological synthesis [3]. However, some problems in the synthesis control and reproducibility could arise if the whole extracts, which may contain different compounds at different concentrations, are used directly in the formation of NPs. For this reason, the biosynthetic formation of NPs using pure compounds found in plants, at a controlled concentration, was tested successfully in the literature [4, 5]. The obtained AgNPs in this way were proposed for biomedical applications due to their antibacterial and anticancer activities [6, 7].

In most cases, the AgNP generation from plant extracts is followed by a cost-effective technique such as the UV-visible spectrometry in order to establish the best reaction conditions. The interaction of the incident light with the surface plasmon of the nanoparticles generates localized surface plasmon resonance (LSPR) which showed a maximum in the range 400–440 nm. The maximum value depends on the size, shape, and composition of AgNPs and the surrounding medium [8]. However, changes in the synthesis conditions can cause the increase or decrease of the absorbance of the absorption maximum, along with the shift of SPR band to lower and higher wavelength, which may indicate that different phenomena are occurring (changes in the size or in the size distribution, aggregation, etc..). This limited and not very specific information provided by UV-visible spectrometry can be complemented with characterization techniques as electronic microscopy (TEM and SEM) to evaluate of the morphology, size, and dynamic light scattering (DLS) in order to determine the hydrodynamic radius. Nonetheless, the microscopic techniques have limitations to distinguish different stages of reaction as the initial nucleation with low number of particles and sizes. In addition, Fourier-transform infrared spectroscopy is used to dilucidate the interaction of NPs with ligands of the capping agent. Therefore, the following of the synthesis is relevant because the AgNP properties and their toxicity are conditioned by established synthesis process [9].

On the other hand, other species are present in the synthesis medium (silver ions, products of oxidation of the reducing agents…), and therefore, information about them may be of interest. Other analytical techniques, such as field flow fractionation (FFF) [10] or some chromatographic techniques, which allow the speciation, can be useful for this purpose. High-performance liquid chromatography coupled to inductively coupled plasma-mass spectrometry (HPLC-ICPMS) has given good results for the separation of silver species (I) and AgNPs of different sizes (10–40 nm [11] or 1–100 nm [12]). The developed methods have been applied to the analysis of textile products [11] and environmental waters [12]. However, the use of liquid chromatography combined with LSPR detection employing UV-vis is still unexplored in this context. Only a few studies which used liquid chromatography with UV-visible detection have accomplished to obtain a response proportional to the AgNP concentration [13–15].

Regarding the analytical approach, the biogenic synthesis has been employed for the determination of some reducing agents, such as polyphenols. These compounds are related to the prevention of diseases originated by oxidative stress; therefore, their analysis is relevant in food samples, to determine the content of polyphenols or the oxidant capacity of the food matrix. Analytical methods based on the generation of AgNPs have been proposed using the reduction of the silver salts by polyphenols and spectrophotometric measurement of LSPR of AgNPs. In some of them, AgNPs have been generated in the presence of capping agents, which stabilized them [16]. Other approaches used the generation of AgNPs in the presence of AgNP seeds stabilized with citrate [17], chitosan [18], and poly(vinyl alcohol) [19]. Therefore, with these methods, the growth of NPs (and not the nucleation) was established as a function of the concentration of polyphenol.

In the present work, the starting hypothesis assumes that the IT-SPME-CapLC system can provide a useful information about changes that may occur at the AgNP nano-environmental level and about the evolution of their hydrodynamic radius [15]. On the other hand, the biogenic synthesis of AgNPs would allow the determination of the reducing agents (Trolox and chlorogenic acid) in the presence of an excess of precursor (Ag) following a strategy similar to that of the classical derivatizations. Thereby, the objectives of this work were (i) studying the in situ biosynthetic generation of AgNPs using Trolox and chlorogenic acid under different synthesis conditions and (ii) quantifying chlorogenic acid in dietary products by IT-SPME CapLC. To the best of our knowledge, the IT-SPME-CapLC system was used for the first time, for examining the effect of the synthesis parameters (molar relation, temperature, addition of surfactants) on the AgNP generation and their evolution over time.

## Experimental

### Instrumentation

The chromatographic system used was a high-performance liquid chromatography system from Agilent Technologies equipped with a LC capillary binary gradient pump (Agilent 1200 Series) and diode array detection. The injection loop was replaced by a 20-cm capillary column of polydimethylsiloxane (PDMS) with 35% polydiphenylsiloxane (TRB-35, Teknokroma), in which an in-tube solid-phase microextraction of analytes was performed.

The generation of AgNPs was monitored by a fiber optic UV-vis spectrophotometer from Varian Cary 60, which recorded the signal from 200 to 800nm. A transmission electronic microscope (TEM) with a JEM-1010 from Jeol Ltd., which operated at 100 Kv, was used for the characterization of AgNPs. For the characterization of the hydrodynamic radius of AgNPs in suspension, dynamic light scattering (DLS) was applied with ZetaSizer Nano series (Malvern) under the following conditions: T=25°C, analysis time 70 s, position of measurement of 4.65 mm, and attenuation 10.

### Reagents

For the generation of AgNPs, the following reagents were used: sodium hydroxide (Scharlau, Spain), tetradecyltrimethylammonium chloride (≥98) (C14TAC), cetyltrimethylammonium chloride (≥98) (CTAC, Sigma), cetyltrimethylammonium bromide (CTAB) (≥98 %, Sigma), tetrabutylammonium bromide (Bu4B) (≥98 %, Sigma), silver nitrate (%, VWR Chemicals), and Trolox (97%) and chlorogenic acid (>95%) from Sigma-Aldrich, Germany. For the preparation of the mobile phase, ammonium acetate (>98%, Sigma-Aldrich, Germany), sodium dodecylsulfate (>85%) and sodium thiosulfate, from Merck, Germany, were employed.

### Chromatographic conditions

The capillary column used was Zorbax-SB-C18 (35 × 0.5 mm × 5 μm, 80 A) and the mobile phase was prepared in water purified through a Barnstead Nanopure II system containing ammonium acetate (10 mM), sodium dodecylsulfate (10 mM), and sodium thiosulfate (1.5 mM). The latter reagent was used to prevent the possible interactions of Ag^+^ species with stationary phase by the complexation of Ag^+^ with thiosulfate. The mobile phase was filtered through 0.20 μm, sonicated for 1 h and kept at rest for a day. The flow-rate was 12 μL min^−1^. The signal was recorded in the range 200–800 nm. The chromatographic conditions have been based on previous studies of the MINTOTA research group [13, 14]. In the present work, 20-cm capillary column in IT-SPME was used with good results.

It was observed that one of the derivative peak (tr=0.85 min) was highly concentrated in the capillary of IT-SPME. Therefore, cleaning with water the capillary after injection and the control of the injected Trolox concentration were necessary in order to avoid memory effects.

### Synthesis of AgNPs using Trolox as a bio-reducing agent

#### *Procedure A*

Silver nitrate concentration was 380 μM, and the TX concentrations were 25, 50, 150, 250, and 500 μM. Thus, 50 μL silver nitrate, 50 μL Trolox, 15 μL NaOH 1M, and 35 μL of water were mixed in an Eppendorf vial. The mixture was injected directly after 10 min of reaction at room temperature. The chromatographic peak with retention time 1.0 min was monitored at 430 nm (Table 1).

**Table 1:** Different conditions of synthesis tested using Trolox and chlorogenic acid as bio-reducing agents. (Procedure A, B, and C monitorized λ=430 nm, procedure D, E, and F monitorized λ=400 nm)

Procedure	[Ag^+^] (μM)	Reducing agent (μM)	Molar relationship	T	Surfactant	Monitored peak, tr (min)/monitoring time, tm
A Trolox	380	[Tx] = 23–500	[Tx]/[Ag^+^] = 0.07–1.3	Room	No	tr=1/tm=10min
B Trolox	380	[Tx] = 25	[Tx]/[Ag^+^] = 0.07 [Ag^+^]/[Tx] =15.2	Room	Yes/no	tr=1/tm=10min tr=0.7/tm= h
C Trolox	25–200	[Tx] = 3	[Ag^+^]/[Tx] = 8.3–66.6	Room/40°C	Yes	tr=0.7/tm=17 h
D Chlorogenic acid	410	[CGA] = 250–3000	[CGA]/[Ag^+^] = 0.6–7.3	Room	No	tr=1/tm=10min
E Chlorogenic acid	50	[CGA] = 100–500	[CGA]/[Ag^+^] =2–10	Room	No	tr=1/tm=10min
F Chlorogenic acid	25–200	[CGA] = 100	[Ag^+^]/[CGA]= 0.25–2	Room	No	tr=1/tm=10min

#### *Procedure B*

Fifteen microliter 80 μM C14TAC, 15 μL NaOH 1M, 50 μL silver nitrate, 50 μL Trolox, and 20 μL of water were mixed in an Eppendorf vial. The chromatographic profile was monitored using 380 μM Ag and 25 μM of Trolox concentration (molar relationship of 15.2) at 430 nm (Table 1).

#### *Procedure C*

The monitoring of synthesis was performed using 100 μM Ag and 3.3 μM of Trolox concentration (molar relationship of 30.3) at different times. The synthesis was performed using the order of addition and volume of reagents according to *procedure B*. Syntheses at room temperature and thermal synthesis (at 40°C in stove) were performed. In the latter case, different types of cationic surfactants (C14TAC, CTAC, CTAB, and Bu4 B) were tested.

For the quantitation of silver, 3.3-μM Trolox concentration and 23, 50, 100, and 200 μM of silver concentration were used. The mixture was injected after 17 h of reaction at room temperature. The chromatographic peak with retention time of 0.7 min was monitored at 430 nm (Table 1).

#### Synthesis of AgNPs using chlorogenic acid as a bio-reducing agent

Three synthesis procedures (*D, E*, and, *F*) were tested. The concentration and molar relationships of silver salt and chlorogenic acid are shown in Table 1 for each of the procedures. Thus, 15-μL NaOH 1M, 50 μL chlorogenic acid, 50μL silver nitrate, and 35 μL water were mixed in an Eppendorf vial. For their injection in the chromatographic system, the solutions were diluted five times. The chromatographic peak with retention time of 0.7 min was monitored at 400 nm.

#### Application to the determination of CGA in dietary products

Commercial samples of green coffee belonging to two different brands were analyzed. Each tablet was dissolved in 250 mL of water (purified through a Barnstead Nanopure II system) according to the recommended dose for the marker (100 mg/250mL for brand 1 and 157 mg/250mL for brand 2). The supernatant was diluted (1:5) to a concentration of 226 μM according to the product specifications. Several dilutions of the samples were prepared (181, 136, and 90 μM).

## Results and discussion

### Formation of AgNPs using bio-reducing agents by IT-SPME-CapLC-UV

The extent on the in situ generation of AgNPs is mainly governed by the molar relationships between the silver salt and the reducing agent (Trolox and chlorogenic acid). The process started with the reduction of the silver salt to metal and the formation of a derivative from polyphenol, containing a quinone structure [17, 18]. Then, the AgNPs grew until their stabilization in the suspension. The basic medium is necessary to deprotonate the hydroxyl group of polyphenol, favoring the reaction and stabilizing the suspension. In the literature, polysaccharides such as chitosan or cationic surfactants [16] or a flavone structure (apiin) on the surface of NP [4] was also used in order to improve stabilization.

**Fig. 1:**
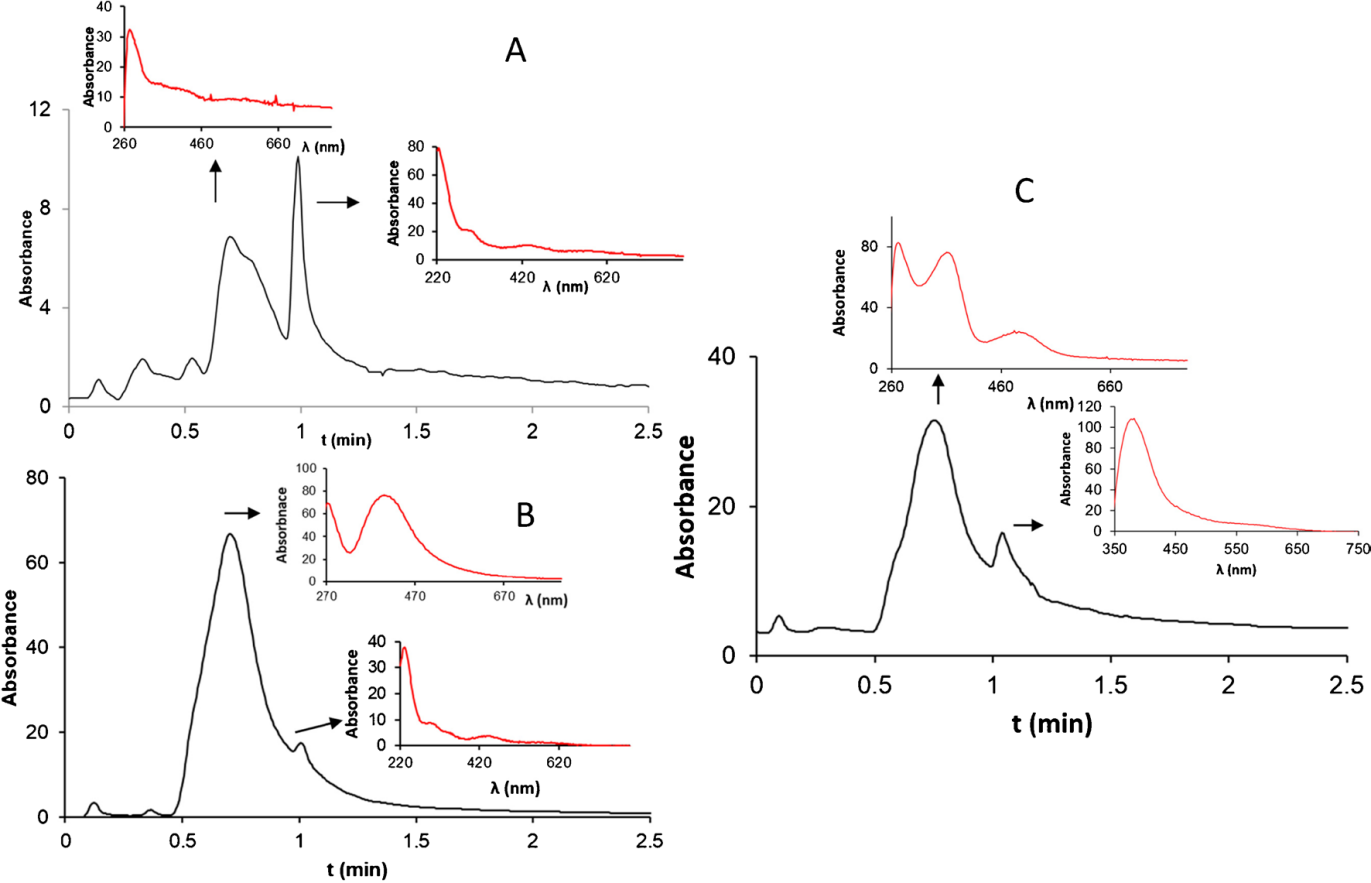
Chromatograms with their corresponding spectra obtained using Trolox as a bio-reducing agent (procedure B with C14TAC) at 10 min of reaction (**A**) and after 17 h of reaction (**B**) (chromatogram at λ= 430nm). Chromatogram using chlorogenic acid (50 μM) (procedure A) at 10 min (**C**) (chromatogram at λ= 400nm)

The study of synthesis conditions was performed using different molar relationships (with excess of the precursor (Ag^+^) or Trolox in order to monitoring of the AgNP formation as well as to examine the analytical possibilities of the synthesis for determining the reducing agents. Three synthesis procedures were evaluated as described in the experimental section and in Table 1, where they were named *procedure A* (with excess of Trolox)*, B* (with excess of silver and fixed molar relation), and *C* (excess of silver, at controlled temperature and using cationic surfactants)*.* Three chromatographic peaks were detected under different tested procedures using the IT-SPME-CapLC-DAD conditions. A chromatographic peak with the retention time of 0.85 min and a spectrum which had a maximum at 270 nm was observed, possibly corresponding to the Trolox derivative [18]. In the wavelength range of LSPR, two chromatographic peaks with retention times of 0.7 min and 1.0 min were detected depending on the tested synthesis conditions (Fig. 1A and B).

**Fig. 2:**
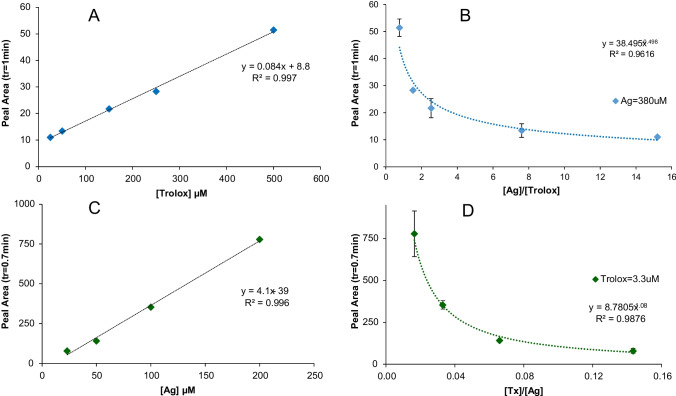
**A** Calibration plot of Trolox by procedure A ([Ag] = 380 μM, 10 min reaction, λ= 430nm, tr=1min). Linear range: 25–500 μM Trolox *n*=8. **B** Relationship between the peak area (tr=1.0 min) and [Ag]/[Tx] using a constant Ag^+^ concentration (380 μM). **C** Calibration plot of Ag by procedure C ([Trolox] = 3.3 μM, 10 min reaction, λ= 430nm, tr=0.7min). Linear range: 25–200 μM Ag *n*=8. **D** Relationship between the peak area (tr=0.7 min) and [Tx]/[Ag] using a constant concentration of Trolox (3 μM)

In *procedure A*, the Trolox concentration was varied in the range from 23 to 500 μM, the concentration of silver salt (380 μM) was constant, and thus, relationships [Ag]/[Trolox] from 0.8 to 15.2 were used (Table 1). The synthesis was monitored at 10 min integrating a chromatographic narrow peak with retention time of 1 min at 430 nm. Another peak at tr= 0.7 min was detected, although the plasmonic band cannot be identified (see Fig. 1A). Under the tested conditions, no increase in the peak area with retention time of 1 min was observed over time although the peak increased with Trolox concentration. A linear relationship between the chromatographic peak area or height and the Trolox concentration was established at 430nm (Table 2 and Fig. 2A). Consequently (since [Ag+] is a constant), a linear relationship between peak area and molar relation [Trolox]/[Ag] was observed (A= 31.97 [Trolox]/[Ag]+8.75, *R*^2^=0.9973). Therefore, these conditions could be employed to quantify the reducing agent. On the other hand, the peak area was related to the [Ag]/[Trolox] molar relationship by means of a potential equation (see Fig. 2B). The minimum of the equation corresponded to an [Ag]/[Trolox] molar relationship = 2.8, indicating the minimum relationship for which the AgNPs started to increase. This estimated stoichiometry could be related to the formation of the Trolox-stabilized nanoparticles [4].

**Table 2:** Relationships between peak area or height and the reagent concentration (silver and reducing agent) under different conditions

**Reducing agent**	**Conditions**	**Equation**				
		***y***	***x***	***a*±*s***_**a**_	***b*±*s***_**b**_	***R***^**2**^
Trolox	Procedure A	Area	[Tx]	8.8 ± 0.7	0.084± 0.003	0.997
		Height	[Tx]	2.8 ± 0.3	0.03± 0.01	0.994
	Procedure C	Area	[Ag^+^]	−39 ± 22	4.1± 0.3	0.996
		Height	[Ag^+^]	−3 ± 1	0.24± 0.01	0.994
Chlorogenic acid	Procedure D	Area	[CGA]	750 ± 419	13.1 ± 0.9	0.9831
		Height	[CGA]	19 ± 5	0.22± 0.01	0.9911
	Procedure E	Area	[CGA]	2320 ± 375	31 ± 1	0.9943
		Height	[CGA]	33 ± 12	0.63± 0.04	0.9855
	Procedure F	Area	[Ag^+^]	3094 ± 378	26 ± 3	0.9547
		Height	[Ag^+^]	65 ± 7	0.35± 0.06	0.9706

In procedure *B*, the highest [Ag]/[Trolox] relationship =15.2 (and highest excess of Ag), 380 μM concentration of silver salt and 25 μM of Trolox were used. The profile of the chromatogram over the time (in hours) was studied. The addition of the cationic surfactant C14TAC, in order to stabilize the formed AgNPs, led to a significant increasing of the first peak area (tr= 0.7 min). After 17 h, the peak area was three times higher using C14TAC than without surfactant. A yellow color could be observed in the solution showing the first peak a spectrum with SPR (maximum around 400 nm) (Fig. 1B). However, the second peak with tr=1.0 min was maintained constant, although for high areas of the peak 0.7 min was partially overlapped in the tail. This fact can be explained if the more retained peak (tr= 1.0 min) corresponded to the AgNPs formed in the first step in which Trolox could have adsorbed on the AgNPs. Kasthuri et al. [4] described the adsorption on the AgNP surface of the derivatives of polyphenols with metal ions, making up a micellar structure. The binding to the metallic ion occurred through the carbonyl group of polyphenol (Fig. 1S). In the case of Trolox, the quinone formed in the oxidation of phenol had a group carbonyl. The stabilization of the Trolox as a derivative stabilized AgNP would explain the fact that it does not suffer any changes over time. However, the peak corresponding to the polarized AgNPs is the one that increased over the time. The growth of the peak (directly related to the absorbance) suggests that the largest population and a smaller size are formed (shift of the maximum to the lower wavelength) [20].

Similarly to procedure *A*, a third biosynthetic procedure (procedure C) was developed maintaining the Trolox concentration constant and varying the silver concentration (Table 1). Thus, higher silver salt/Trolox molar relationships (7 to 60) with a low concentration of Trolox (3.3 μM) and using C14TAC for NPs stabilization were tested. Under these conditions, a linear relationship was found between the area and the height of the chromatographic peak (tr= 0.7 min) and the silver concentration (Table 2). From the point of view of the analytical application, this relationship could be used for the quantitation of silver by the green synthesis of AgNPs (Fig. 2C). As can be expected, a linear relationship between the peak area and [Ag]/[Trolox] can be established (A= 13.35 [Ag]/[Trolox] − 39.28, *R*^2^=0.9956) at 430nm. A potential relationship between the peak area and [Trolox]/[Ag] was found (Fig. 2D). In this case, the estimated minimum of the equation corresponded to 24.7 = [Ag]/[Trolox] for which the growth of the second kind of the generated AgNPs started. An [Ag]/[Trolox] relationship higher could suggest that there was more Ag^+^ on the surface and therefore more polarized AgNPs that eluted earlier from the chromatographic system.

**Fig. 3:**
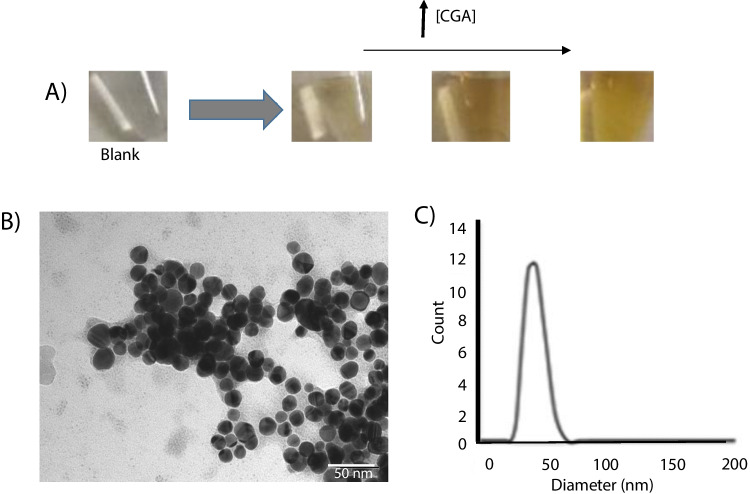
**A** Changes of color due to an increase in the chlorogenic acid concentration in the syntheses of AgNPs. **B** Measurement of the hydrodynamic radius of AgNPs by DLS. **C** Photograph of AgNPs by TEM

Finally, the monitoring of the AgNP generation was performed at 100 μM silver concentration, an [Ag]/[Tx] molar relationship of 30 and at room temperature. The peak area (tr=0.7 min) was increasing over time, and after 17 h (staying at rest overnight), the solution developed a yellow color. The increase of the peak could be interpreted as an autocatalytic surface growth (Fig. 2S) [21]. The reduction of Ag^+^ occurred on the surface of the previously formed AgNPs via electron transfer. This fact could be explained by the fact that the peak area was proportional to the silver concentration. The Finke–Watzky model explained this mechanism in which nucleation and growth occurred simultaneously (more details are given in the section 2 of the Supplementary Information) [20, 22]. The effect of the temperature on the synthesis of AgNPs is discussed in section 3 of the Supplementary Information (Fig. 3S).

The effect of the cationic surfactant (alkyltrimethylammonium type) on the generation of AgNPs was also examined. The molecules of the cationic surfactants were adsorbed on the surface of NP due to the electrostatic interaction (positive charge of the surfactant and negative surface charge of NP), providing a certain degree of hydrophobicity to the AgNPs [8, 23, 24]. Thereby, several surfactants with differences in counterion (chloride or bromide) and/or in the length of the longest alkyl chain (tetradecyl- or hexadecyl-) were tested (CTAC, CTAB, C14TAC, Bu4B). Fig. 4S shows the results normalized for the peak area (tr=0.7 min) when hexadecyltrimethylammonium chloride (CTAC) was used in the thermal synthesis. A decrease in the alkyl chain length from C16 to C14 using C14TAC yielded 80% of the area obtained with CTAC. A better stabilization of NPs by increasing the length of the alkyl chain was described in the literature [24]. A greater effect was caused by the change of anion (bromide and chloride); see CTAC versus CTAB. It is worth noting that some inorganic anions as sulfide, chloride, borate, and carbonate can also act like capping agents [19]. The anion of surfactant can determine the NP shape, nanorods using CTAB, and spherical NPs using CTAC were reported by Murphy et al. [25]. In the present work, there was no evidence of this change of shape. The anion could form silver halide and adsorb on the surface of NPs and could also decrease the reduction of silver [26]. A cationic surfactant with the shorter alkyl chain and with bromide anion (Bu4b) obtained the worst results and the highest dispersion. It should be noted that without surfactant (only water), the area was 60% of that obtained using CTAC.

### Application to dietary products

Initially, procedure *A* proposed for Trolox was tested with chlorogenic acid, obtaining a chromatogram with two peaks (0.7 and 1 min). However, there were differences in the UV spectra in peak apex between them and those obtained using Trolox (see Fig. 1C). The spectrum of the first peak showed two bands. Zhong et al. [23] described a similar phenomenon for AuNPs of the same shape (spherical) and size (20 nm) but different degrees of aggregation. The first band of the spectrum has a maximum near the characteristic wavelength for single particles (it corresponds to quadrupole plasmon to coupled spheres), and the other was displaced to longer wavelengths (it corresponded to dipole plasmon resonance). Although this may suggest that the first peak could contain nanoparticles with different degrees of aggregation, the effect of CGA or its derivatives (product of coordination with metal or quinones) on the surface of AgNPs or between the inter-particle spaces would not be negligible. A shift to shorter wavelengths for two bands to increase CGA concentration was observed. Thereby, for one of the bands, the maximum values were 484nm for 50 μM and 464nm for 500 μM of CGA. If the CGA or quinone derivative covered the AgNPs with a thinner layer (a lower concentration), a shift to longer wavelength would occur.

Unlike the syntheses using Trolox, the two chromatographic peaks responded to the CGA concentration. In the comparison between both compounds, one has to take into account the two functions as reducing and stabilizing agents. The higher reducing power of CGA than Trolox may be due to two hydroxyl groups on the aromatic ring of CGA versus one group of Trolox [22]. Polar groups such as the carboxylate group presented in two compounds could complex ion silver and form a coat on the AgNP. This coating stabilized AgNPs and led to their growth. Other authors verified the coordination of CGA by -C=O in AgNPs by Fourier-transform infrared spectra [27].

On the other hand, a different strategy of synthesis was also adopted using chlorogenic acid. Room temperature, without cationic surfactant for stabilization, and low molar relationships between the reagents (silver and chlorogenic acid) were employed for the synthesis. Higher concentrations of the reducing agent than in synthesis with Trolox and dilution of AgNPs before chromatographic injection were other different tested conditions.

The effect of silver and CGA concentration on AgNPs was examined (Table 1). In procedure D, the silver concentration (410 μM) was maintained constant, chlorogenic acid concentration being varied in the range from 250 to 3000 μM. Under these conditions, a linear relationship between the second peak area and the concentration of chlorogenic acid as well as a [chlorogenic acid]/[silver] molar relationship was found (Table 2). For the quantitation of the chlorogenic acid, in procedure E, a lower silver concentration (50 μM) and of chlorogenic acid (100–500 μM) were used, keeping similar molar relationships as procedure D. As shown in Table 2, a higher slope was achieved using 50 μM than 410 μM.

A higher slope of calibration plot and, therefore, higher sensitivity were obtained using chlorogenic acid (procedure D) than using Trolox (procedure A) with the same silver concentration (410 μM), although the molar relationships were also higher in procedure D. The high number of -OH groups determined a higher efficiency of CGA reduction than Trolox [22]. The effect of silver concentration on AgNPs using a CGA concentration of 100 μM (Table 2) is discussed in section 5 of Supplementary Information.

The formation of AgNPs can be followed by a high intensity of yellow color. The size of AgNPs was determined by TEM and the dispersion light scattering (DLS), obtaining values of 20 nm and 34 nm (hydrodynamic diameter), respectively (Fig. 3). Other analytical parameters as limit of detection and precision were estimated. LOD was calculated as 3 sb /b, sb being the standard deviation of blank, and b the slope of calibration plot obtaining 34 μM. The precision was expressed as a variation coefficient for which *n*=3 was 10%.

Two commercial dietary products (green coffee A and B) containing chlorogenic acid according to the manufacturer specifications were analyzed with the proposed method. Each sample was diluted up to concentrations, which were within the linear range. The generation of AgNPs was performed using a silver concentration of 50 μM. The linear function between the peak area and the expected concentrations in the samples for the two products are shown in Table 3. The % recovery of chlorogenic acid in the samples was calculated by the relationships between the slope of the calibration plot standards and the slope obtained for the sample (*b*_standard_/*b*_sample_) considering the concentration given by the manufacturer. The results were 100% for sample A and 97% for the sample B, and then, no matrix effect was found. Therefore, the found values were 157±16 CGA/tablet for sample A and 97 ± 10 mg CGA/tablet for sample B.

**Table 3:** Linear relationships between peak area in the samples and the expected concentrations (*n*=3)

*y* = *a* + *bx*			
*x*	*a*±*s*_a_	*b*±*s*_b_	*R*^2^
Sample A	−639 ± 694	30 ± 3	0.976
Sample B	−1803 ± 467	31 ± 2	0.994

On the other hand, procedure *A* for Trolox was applied to standard solutions of chlorogenic acid and a commercial sample of green coffee. Two peaks with an area proportional to the concentration of CGA in the range from 50 to 3000 μM were found. The profile, with a first major peak, was similar to those obtained using procedures of Trolox in which the silver was in excess. Under these conditions, the sample of green coffee (diluted to 30%) was processed, and the evolution of peaks was monitored over time. Three peaks were found, out of which the first and the third increased over time (10, 42, and 72 min) (Fig. 6S). In addition to CGA, other polyphenols contained in the formulation of coffee could generate AgNPs.

### Comparison with other methods reported in the literature

Table 4 summarizes the characteristics of the selected methods for the determination of CGA in various food matrices employing chromatographic, spectroscopic, and electrochemical techniques. The spectroscopic methods, which have as principle the formation of AgNPs, measured the total antioxidant activity (TAC) since different kinds of polyphenols contained in the samples exhibited reductor potential with silver salts under optimized conditions [16, 17]. In our work, the time of AgNP synthesis was lower than time optimized in these methods. In addition, some methods employed several stages of sample treatment increasing the analysis time. It should be noted that the obtained results were comparable than those obtained with the established methods for the determination of TAC. In the present work, this option has not been considered although the qualitative application to samples of the procedure A (Figure 5S) suggests that TAC could be measure as the sum of the areas of chromatographic peaks. Other methods exhibited good LODs as those that used a fluorometric probe (0.045 μM) [28] or voltammetric sensors (0.06 μM) [29]. In our method, LOD was not a critical analytical characteristic due to the high concentration levels of CGA in the samples. In the method proposed by Munteanu and Apetrei [29], a study of redox process was carried out in addition to analytical application. In the same way, the present work approached both the AgNP synthesis and its application to CGA determination. On the other hand, the main isomers of CGA have been determined using miniaturized chromatographic techniques and conventional HPLC for their application in quality control due to portability [30] or robustness [31]. In the present work, the objective was focused in the major isomer (5-caffeoylquinic or chlorogenic acid) accomplishing a more rapid method with a wide linear range and a possible application to the TAC determination. The chromatographic profile supporting by chemometrics tools (e.g., that obtained using procedure A) could be used in order to discriminate between samples from different commercial sources.

**Table 4:** Comparison with other reported methods for the determination of CGA using chromatographic, spectroscopic, and electrochemistry techniques

Matrix	Steps and time	Technique	Linear range (μM)	LOD (μM)	% RSD	Remarks	References
Tea and different kinds of infusions	◼ Extraction (1h), centrifugation (10min) and filtration ◼ Formation of AgNPs (20–22 min) ◼ UV-visible spectrum (TAC*)	UV-visible	CGA 3–25 20–90	Gallic acid (reference) 0.4–34	3–14	Study of the reactivity. TAC* due to CGA and other 14 polyphenols. Comparison with the established methods High analysis time	[16]
Fruit juices and herbal teas	◼ Formation of AgNPs (30 min) ◼ UV-visible spectrum (TAC)	UV-visible	CGA 1.3–49.8	0.2	0.3–2.6	TAC due to CGA and other polyphenols. Comparison with the established methods. High time for the formation NPs	[17]
Honeysuckle	◼ Ultrasonic extraction (30 min) ◼ Dilution and filtration ◼ Dilution and mixture with CD ◼ Fluorometric measurement (5min)	Fluorometric probe (carbon dots, CD)	0.15–60	0.045	2.2–3.8	Sample treatment with several stages. Very good LODs. It is necessary the preparation and characterization of the nanomaterial	[28]
Dietary products (green coffee)	◼ Dilution ◼ Sonication and filtration ◼ Measurement	Voltammetric sensors (Screen printed electrodes)	0.1–1.2	0.06	2.5	Study of the oxidation and reduction process. Very sensitive method. Narrow linear range	[29]
Dietary products (green coffee)	◼ Ultrasonic extraction (6min) ◼ Filtration ◼ Dilution and acidification ◼ Chromatogram (6–24 min)	Portable LC CapLC Nano-LC	85–226** 0.6-6 0.03–2.8	14** 0.14 0.014	1** 2 5	Separation of the CGAs profile (6 compounds). Very good LODs using Nano-LC. Higher analysis time in Cap and Nano-LC than portable LC	[30]
Green coffee beans	◼ Supercritical/methanolic extraction ◼ Dilution ◼ Sonication (10 min) ◼ Chromatogram (19min)	HPLC	27–3666	3.2 (LOQ)	0.6–2	Separation of the CGAs profile (7 compounds). Validation in three independent laboratories. Several treatment stages after to obtain the extract	[31]
Dietary products (green coffee)	◼ Dilution ◼ Formation of AgNPs and dilution (10min) ◼ Chromatogram (3 min)	IT-SPME-CapLC	250–3000	34	10	Study of the formation of AgNP nanoparticles. Wide linear range. Short analysis time. Higher LOD than other chromatographic methods	This work

*TAC total antioxidant activity. ** Values for 5-CGA

## Conclusion

IT-SPME-CapLC-DAD allowed the monitoring of AgNPs under different conditions of synthesis. The two detected chromatographic peaks had different spectra for each, different behavior while monitoring the synthesis and with the reagent concentration, indicating that two different coated AgNPs were separated. Therefore, IT-SPME-CapLC-DAD is able to provide information on the synthesis procedure, which would be difficult to obtain using other techniques. The increase over time of the more polarized AgNPs suggested an autocatalytic growth. A thermal synthesis with excess of silver increased the velocity of the reaction. Both the alkyl chain length and the counterion of the cationic surfactant (used as stabilizing agent) had effect on the formation of AgNPs. A longer alkyl chain and chloride ion favored a greater increase of the chromatographic peak than a shorter chain and bromide ion.

The chlorogenic acid, as expected due to its chemical structure, showed higher reactivity than Trolox for the generation of AgNPs. Syntheses using excess of both silver and chlorogenic acid/Trolox allowed obtaining an analytical signal (peak area and height) proportional to both silver and chlorogenic acid/Trolox concentration. Based on the studied syntheses, a method was proposed for the determination of chlorogenic acid in samples of green coffee in a short analysis time (13 min) and with a wide linear range and excellent recoveries. Although the proposed method has higher LOD than other chromatographic methods, it could be applied to determine the total antioxidant activity in dietary products contained several types of polyphenols. Furthermore, considering the results, the discrimination of samples from different commercial sources could be addressed in future works.

## Supplementary information

ESM 1
