# Supplementary material for: Determination of antioxidant activity by in situ synthesis of AgNPs using in-tube SPME coupled on-line to capillary liquid chromatography
Source: Mikrochim Acta. 2023 Jul 18;190(8):299. doi: 10.1007/s00604-023-05886-w (PMC10353952; doi:10.1007/s00604-023-05886-w)
Supplement: Supplementary file 1 — ESM 1 [file 604_2023_5886_MOESM1_ESM.docx]

**Electronic Supplementary Material**

**DETERMINATION OF ANTIOXIDANT ACTIVITY BY IN-SITU SYNTHESIS OF AgNPs USING IN-TUBE SPME COUPLED ON-LINE TO CAPILLARY LIQUID CHROMATOGRAPHY**

M.C. Prieto-Blanco^1*^, M. Pardo-Puñal^2^, Y. Moliner-Martínez^2^, P. Campíns-Falcó^2^

^1^Universidade da Coruña, Grupo QANAP, Instituto Universitario de Medio Ambiente (IUMA), Departamento de Química, Facultade de Ciencias, Zapateira, 15071, A Coruña, Spain

^2^Grupo MINTOTA, Departament de Química Analítica, Facultat de Química, Universitat de Valencia C/ Dr. Moliner 50, E46100- Burjassot, Valencia, Spain

*Corresponding author: [m.c.prieto.blanco@udc.es](mailto:m.c.prieto.blanco@udc.es)

**Section 1.** **Structural formula**

The structural differences between the two bio-reducing agents are shown in Fig, 1S

**Fig. 1S** Structural formula of the bio-reducing agents.

**Section 2.** **Mechanism of autocatalytic reduction-nucleation**

**Fig. 2S** Generation of AgNPs using phenolic compounds. Growth of AgNPs according to the mechanism proposed by Harada and Katagiri [1].

Jana et al., [2] demonstrated this mechanism for the formation of gold nanoparticles by other compound with hydroxyl groups (ascorbic acid). Harada and Katagiri, [1] also found an autocatalytic reduction-nucleation of AgNPs in PVP, using photoreduction in presence of benzoin (their hydroxyl groups were oxidized in this process). These authors observed an increase in average radius and number of nanoparticles (nucleation process) during the first 10 minutes, and afterwards an increase in radius and a decrease in the particle number. In the present work, the CapLC did not allow distinguishing whether the increase in the peak area was due to the increase in radius and/or number, although a shift in the maximum of SPR was not observed.

**Section 3. Effect of the temperature on the synthesis of AgNPs**

In order to examine the effect of the temperature on the synthesis of AgNPs, the procedure *C* was developed at 40ºC without agitation. An increase in reaction velocity was achieved since the peak area after 90 min was 1.2 times that obtained after 5 hours at room temperature. An evolution over time of the thermal synthesis is shown in Fig. 3S. If this solution was kept at room temperature, the formation of AgNPs continued to occur up to 2 days and the peak area remained stable for at least five days.

**Fig. 3S** Evolution over time of the thermal synthesis of AgNPs using Trolox as a bio-reducing agent.

**Section 4. Effect of surfactant type**

**Fig. 4S** Effect of surfactant type on the peak area (tr= 0.7 min) in thermal synthesis (normalized by CTAC).

**Section 5. Synthesis procedure *F* using chlorogenic acid**

On the other hand, the effect of silver concentration was studied in the procedure *F* keeping constant the concentration of CGA (100 µM). In this case, without a high silver excess, the peak which responded to the concentration of Ag was the second one. A linear relationship between the peak area or height and silver concentration could also be established (Table 2).

Similarly to Trolox procedures, potential equations were established between the peak area and [Ag]/ [CGA] for the procedures D and E. At 410 µM of Ag, the estimated minimum corresponded to [Ag] / [CGA] =0.24 for the growth of the second peak (Fig. 5S A). Unlike Trolox ([Ag]/ [Tx] =2.8), a greater excess of reducing agent would be necessary to start the growth, possibly due to a different adsorption of polyphenol on AgNPs. Likewise, a potential equation was established in the procedure F (peak area versus [CGA]/[Ag]/) (Fig. 5SB).

**Fig. 5S A)** Relationship between the area peak (tr=1.0 min) and the [Ag^+^]/[CGA] molar relationship using [Ag^+^] = 410 µM and [Ag^+^] = 50 µM **B)** Relationship between the area peak (tr=1.0 min) and the [CGA]/[Ag] molar relationship using [CGA] = 100 µM.

**Section 6. Application of the procedure A to samples**

**Fig. 6S** Chromatogram of a green coffee sample over time using procedure A. Orange line=10min, green line= 42min, blue line=72 min.

**References**

1. Harada M, Katagiri E (2010) Mechanism of silver particle formation during photoreduction using in situ time-resolved SAXS analysis. Langmuir 26(23):17896–17905. <https://doi.org/10.1021/la102705h>
2. Jana N R, Gearheart L, Murphy C J (2001) Evidence for seed-mediated nucleation in the chemical reduction of gold salts to gold nanoparticles. Chem Mater 13:2313-2322. <https://doi.org/10.1021/cm000662n>
